# Gastrointestinal Stromal Tumours: An Update

**DOI:** 10.1080/13577149877885

**Published:** 1998-12

**Authors:** Nicolas De Saint Aubain Somerhausen, Christopher D. M. Fletcher

**Affiliations:** 1 Department of Pathology Brigham and Women's Hospital and Harvard Medical School 75 Francis Street Boston MA 02115 USA; 2 Institut Jules Bordet Université Libre de Bruxelles Bruxelles Belgium

## Abstract

*Purpose.* To study the evolution of concepts concerning gastrointestinal stromal tumours (GISTs) over 30 years.

*Discussion.* GISTs have been, for more than 30 years, the subject of considerable controversy regarding their line of differentiation as well as the prediction of their behaviour. Furthermore, once they spread within the peritoneal cavity, they are extremely hard to control. The recent findings of *c-Kit* mutations and the immunohistochemical detection of the product of this gene, KIT or CD117, in the mainly non-myogenic subset of this family of tumours, has led to a reappraisal of this group of lesions, which, with some exceptions, is now thought to be derived from the interstitial cells of Cajal, and this has facilitated a clearer definition of their pathological spectrum. In this article, we review chronologically the evolution of the concept of GIST with the gradual application of electron microscopy, immunohistochemistry, DNA ploidy analysis. We discuss the impact of these techniques on the pathological assessment and clinical management of GISTs.


Sarcoma (1998) 2, 133–141

REVIEW

### Gastrointestinal stromal tumours: an update

NICOLAS DE SAINT AUBAIN SOMERHAUSEN[1,2] & CHRISTOPHER D. M. FLETCHER[1]

[1]*Department of Pathology, Brigham & Women's Hospital and Harvard Medical School, Boston &* [2]*Institut Jules Bordet, Université Libre de Bruxelles, Bruxelles*

**Abstract**
*Purpose.* To study the evolution of concepts concerning gastrointestinal stromal tumours (GISTs) over 30 years.
*Discussion.* GISTs have been, for more than 30 years, the subject of considerable controversy regarding their line of differentiation as well as the prediction of their behaviour. Furthermore, once they spread within the peritoneal cavity, they are extremely hard to control. The recent findings of *c-Kit* mutations and the immunohistochemical detection of the product of this gene, KIT or CD117, in the mainly non-myogenic subset of this family of tumours, has led to a reappraisal of this group of lesions, which, with some exceptions, is now thought to be derived from the interstitial cells of Cajal, and this has facilitated a clearer definition of their pathological spectrum. In this article, we review chronologically the evolution of the concept of GIST with the gradual application of electron microscopy, immunohistochemistry, DNA ploidy analysis. We discuss the impact of these techniques on the pathological assessment and clinical management of GISTs.

**Key words**: *stomach, small intestine, leiomyoma, tumour, sarcoma.*

### Introduction

Neoplasms arising from the stromal (or mural) components of the gut can be broadly divided into two categories. Some tumours are not unique to the gastrointestinal wall and appear similar to their counterparts in other locations. This category includes schwannomas, usual leiomyomas (mostly located in the oesophagus and rectal wall) and leiomyosarcomas, showing characteristic morphological, immunohistochemical and ultrastructural features of smooth muscle differentiation, as well as some uncommon neoplasms such as lipomatous and vascular tumours. The other category is composed of spindle and epithelioid neoplasms histologically resembling smooth muscle tumours but either lacking or presenting only limited immunohistochemical and ultrastructural features of myogenic, neural or neuronal differentiation, for which prediction of behaviour has proved to be problematic.

Stromal tumours of the gastrointestinal tract (GIST) occur over a wide age range but affect predominantly middle-aged and elderly individuals, with a slight female predominance. Bleeding is the most common initial symptom, and up to 20% of patients present with anemia. Pain represents another common complaint. Despite their large size, only a small proportion of tumours are palpable.[1] The biological behaviour of GISTs is difficult to determine accurately from data available in the literature, given the different morphological and diagnostic criteria used, and the tendency for late metastases, in some cases, with spread sometimes occurring after 20 to 30 years.[2] The overall 5 and 10-year survivals of malignant GISTs, have been estimated at between 25 and 50%,[3,4] although in one series, only 10% of patients remained free of disease after a median follow-up of 68 months.[3] Most such patients succumb to disseminated intraabdominal disease (with metastasis to the omentum, mesentery, peritoneum and liver), although distant metastases (mainly to the lungs and bone) occasionally occur.[5,6] Treatment of GIST is essentially surgical and the type of operation has been shown to represent one of the most important determinants of survival.[3] Surgical excision of intraabdominal metastases has also been shown to slightly improve survival.[6] Unfortunately, GISTs tend to respond poorly to chemotherapy, being even less chemosensitive than leiomyosarcomas at other sites.[7,8]

Despite their relative rarity when compared to epithelial tumours in this anatomical location, these tumours, usually grouped under the non-committal term of gastrointestinal stromal tumour (GIST), have been one of the most controversial subjects in

Correspondence to: Dr Christopher D.M. Fletcher, Department of Pathology, Brigham and Women's Hospital, 75 Francis Street, Boston, MA 02115, USA. Tel: 617 732 8558; Fax: 617 566 3897.

1357-714X/98/030133-09 $9.00 © 1998 Carfax Publishing Ltd the recent pathology literature. Until this year, the large number of studies generated by the progressive use of electron microscopy, immunohistochemistry, flow cytometry and proliferation markers have mostly provided contradictory or inconclusive results regarding the 'histogenesis' of these tumours and determination of their prognosis.

The recent identification of mutations of the *c-Kit* gene in GISTs,[9] and the immunohistological detection of its product, KIT or CD117,[9–11] suggesting differentiation towards the phenotype of the interstitial cell of Cajal,[9,10] has served as the basis for a new definition of GISTs, and has provided an elegant explanation for the previously controversial results relating to the phenotype of these tumours, as well as a new springboard for future investigations.

In this article, using a chronological approach, we review the evolution of the 'concept' of GISTs, from Stout's early reports to the most recent studies, mentioned above, and discuss the impact of these developments on our understanding of GISTs, based on the literature and our personal experience.

### Histogenesis/differentiation

GISTs were originally thought to arise from mural smooth muscle of the gastrointestinal tract, based on their histological resemblance to leiomyomas and leiomyosarcomas at other sites, as well as their usually intimate association with the wall of the gut.[12–14] Epithelioid tumours, first identified by Martin *et al.*[15] and subsequently popularised by Stout,[16] were also considered to be smooth muscle neoplasms on the basis of the transition between spindly and epithelioid areas in some lesions. Subsequently, some authors noticed morphological differences between these 'leiomyomas' and 'leiomyosarcomas' of the gastrointestinal tract and their counterparts in other sites: most gastrointestinal lesions appeared more cellular and the tumour cells had more elongated nuclei and less brightly eosinophilic cytoplasm. However, most authors attributed this to a relative lack of differentiation, rather than to the possibility of alternative lines of differentiation. In addition, it rapidly became clear that GISTs, unlike their apparent counterparts in other sites, could metastasise despite the absence of usual histological features of malignancy (in particular, in the absence of significant mitotic activity) and that their behaviour was much harder to predict.[12,14,17]

### *Ultrastructural studies*

The 1970s and the early 1980s saw the debate concerning these lesions focusing on the ultrastructural features of gastrointestinal sarcomas, of which the presumed smooth muscle differentiation had started to be questioned. In 1969, in their ultrastructural study of three "gastric cellular leiomyomas", Welsh and Meyer noticed that ultrastructural features of smooth muscle (i.e. cytoplasmic filaments with dense bodies, extracellular basement membrane, pinocytic vesicles) were identified in only occasional cells and were often incomplete.[18] These results were confirmed by most subsequent studies, which failed to identify myofilaments with focal densities in most tumours; in fact, smooth muscle differentiation in gastrointestinal stromal tumours was most often supported only by relatively non specific features (such as the presence of pinocytic vesicles or focal basal lamina).[19–23] In addition, several authors started to identify Schwannian or neuroaxonal characteristics, in some cases microscopically indistinguishable from other GISTs,[21,22,24] and in 1984, a distinctive subset of tumours, showing features of autonomic neural differentiation was first described.[25]

### *Immunohistochemical studies*

The introduction of immunohistochemistry, in the 1980s, strengthened the debate relating to the differentiation of GISTs and initiated a profusion of publications.[22,26–34] Numerous series of GIST have been decorated with diverse antibodies with inconsistent results. Although a large proportion (between 30 and 80%) of GISTs have been shown to express muscle markers,[29,33,34] the most specific of these, desmin, has usually stained only a minority of tumours.[30,33] Variable proportions of tumours with a neural phenotype have been identified (from none to approximately 40%)[29,30,33] and, interestingly, divergent differentiation (coexpression of muscular and neural markers) was identified in up to 20% of cases by Newman *et al.*[29] Up to 41% of tumours have been characterised by a 'null' or 'uncommitted' phenotype, being stained by vimentin only.[27] More recently, CD34, initially identified as a myeloid cell progenitor antigen, but also expressed in endothelial cells, in some mesenchymal cells as well as in a variety of soft tissue neoplasms, has been shown to stain up to 80% of GISTs, with or without markers of other specific differentiation.[35–37]

Several reasons may explain the striking lack of consistency of these results, which has rendered their interpretation particularly difficult. Technical issues, relating to the nature of fixative, duration of fixation, nature and dilution of antibodies are certainly partly responsible for some of these discrepancies. Variable thresholds for positivity may have been used and the presence of normal neural elements or muscle bundles may have caused some problems in interpretation. For example, in Mazur's study, first reporting S100 protein immunopositivity in GISTs, seven of eight S100-positive tumours contained only scattered elements which may be more in keeping with entrapped structures than with true nerve sheath differentiation.[22] Variable criteria for smooth muscle and neural differentiation have also been used; for example, while some authors have assessed neural differentiation in GISTs using only antibodies for S100 protein, others have employed a combination of more or less specific markers including NSE, PGP9.5 or Leu7.

Several authors have tried to correlate the immunophenotype of GISTs with tumour location, histological appearance and, more importantly, with prognosis. No significant differences have been demonstrated in terms of immunophenotype between epithelioid and spindle cell lesions. In fact, no reliable correlation between the histological features of GIST and their immunophenotype has been achieved.[27,29] Regarding the relationship with tumour site, the observation that, in contrast to most gastric and intestinal tumours, oesophageal and rectal tumours frequently fail to stain for CD34 and tend to express desmin, has represented an interesting finding, indicating that these tumours likely represent 'true' smooth muscle tumours that should be differentiated from GISTs, which rarely occur in these locations. Some differences in immunophenotype between gastric and intestinal tumours, the latter tending to more commonly express a neural phenotype, have also been suggested[29,33,38] but have not been further investigated.

The possible relationship between the immunophenotype and prognosis has represented one of the more controversial issues in GISTs. Results of a few studies have suggested the possibility of differences in prognosis between immunophenotypic subsets: it has been proposed that tumours with a neural[4,29] or smooth muscle[29] phenotype tend to have a better prognosis, while those with a null phenotype seem more often to behave in a malignant fashion.[34] However, these results have not been confirmed and this field of investigation has gradually been abandoned, on the basis of the consistently inconclusive and variable results. In consequence, many pathologists have stopped phenotyping GISTs in their routine practice. We believe that in the absence of definitive results, the line of differentiation should continue to be part of the information provided to the clinician in any case of GIST and to be included in future studies, until definitely proven irrelevant (or otherwise). In larger studies, the phenotype of GISTs might not only prove to carry some prognostic significance but could also possibly show some relation with treatment response, as, for example, it is well known that leiomyosarcomas at other sites do not usually respond well to chemotherapy.

### *Gastrointestinal autonomic nerve tumours (GANT)*

Among the spectrum of GISTs, specific attention has been given in recent years to those showing autonomic neural differentiation. These tumours were described in 1984 by Herrera *et al.*[25] as 'plexosarcomas', and subsequently designated gastrointestinal autonomic nerve tumour (GANT).[39] Although they tend to be composed of syncytial sheets of cells characterised by distinctive fibrillary eosinophilic cytoplasm, commonly associated with stromal lymphocytes and extracellular nodules of eosinophilic material known as 'skeinoid fibers',[40] their histological spectrum is wide and it is largely accepted that accurate diagnosis is based on ultrastructural criteria, i.e. long cytoplasmic processes with rudimentary cell junctions and synapse-like structures containing dense-core granules.[41] Immunohistochemically, these lesions often show positivity for NSE, usually in a peculiar 'zoning pattern' and, interestingly, they are generally negative for CD34[42] (personal observations). Although these tumours have been the subject of an increasing number of publications,[25,39,41–48] the relative frequency of GANT and its relationship with other GISTs still requires clarification. At the present time, there is no convincing evidence that these tumours differ significantly from other GISTs in terms of either their clinical presentation or behaviour. Nevertheless, further investigations will be required to determine if criteria for malignancy can be established and whether they differ from those applied in other GISTs.

### Prediction of behaviour

The difficulty in classifying GISTs into benign and malignant categories has been recognised since the description of 'smooth muscle tumours' of the gut, by Golden and Stout in 1941, who noted that tumours showing usual histological criteria of malignancy did not consistently behave aggressively, while occasional well differentiated low-grade lesions gave rise to metastases.[12] In a series of 87 GISTs, Kempson and Ranchod identified the mitotic count as the most useful indicator of malignancy. However, while the presence of 5 or more mitoses per 10 HPF was closely correlated with aggressive behaviour, 40% of 'leiomyosarcomas' had fewer mitotic figures.[14] In order to refine the separation of benign and malignant GISTs, a profusion of studies subsequently analysed the correlation between malignancy and various clinical and pathological parameters, often with variable results.[1,4,29,32,34,49–52]

The mitotic count has been most widely accepted as the best prognostic indicator[1,5,14] and it has been shown that, among clinically malignant tumours, a high mitotic count was associated with a shorter disease free interval and shortened overall survival.[5] Various cutoff levels, separating GISTs into benign and malignant categories, sometimes including a 'borderline category', or into low and high-grade subsets have been proposed.[5,51,53,54] However, because of the overlap in terms of mitotic activity between clinically benign and malignant GISTs, and in view of the rare occurrence of metastasis in histologically bland, mitotically inactive tumours, none of these has proven entirely reliable in the management of individual patients.

The extent of disease at diagnosis (or stage) certainly also represents a strong indicator of outcome. While the presence of metastases at presentation, not surprisingly, is associated with a very poor prognosis, infiltration of adjacent structures, such as the liver, pancreas or diaphragm, is also usually regarded as indicative of malignancy. In Shiu's study, all tumours which invaded adjacent organs led to the patient's death.[50] Tumour size has also been shown to be strongly correlated with the occurrence of metastases. In Appelman's series of 127 cases, only one tumour smaller than 6 cm metastasised.[17] These results have been confirmed by most studies and, again, various cutoff levels have been proposed, usually being set at around 5–6 cm. Unfortunately, as for the mitotic count, some exceptions have been encountered, and, tumours as small as 2 cm have been reported to metastasise.[5] In relation to tumour size, it is interesting to note that tumours found incidentally during operation performed for another unrelated condition usually carry an excellent prognosis; in the study of Cooper *et al.* none of 19 incidentally discovered tumours resulted in the patient's death.[51]

In order to refine this discrimination between malignant and benign lesions, numerous other clinical, macroscopic and histological parameters have been assessed, most of which have been shown, at least in some univariate studies, to have some correlation with survival or the development of metastases. Cellularity has been considered useful by several authors,[4,12,14,38,51,55] but this is extremely subjective and difficult to quantitate, and thus is subject to a significant interobserver variability. Moreover, its interpretation is complicated by the variability between areas of the same tumour. Although the presence of unequivocal tumour cell necrosis is usually regarded as highly suspicious for malignancy, this has been reported in rare clinically benign cases.[55] Ulceration of the overlying mucosa has also been considered as a worrisome feature by some authors.[38,55,56] The presence of atypical mitoses has been shown in some studies to be strongly associated with malignancy[52] but the utility of this feature is limited by the fact that abnormal mitotic figures are rarely encountered in GISTs to the point that, in our experience, a diagnosis of GIST is improbable in the presence of conspicuous abnormal mitoses.

Potential differences in terms of behaviour between epithelioid and spindle cell lesions has been another controversial topic; while most studies have not shown any correlation between cell type and prognosis, a few recent studies have suggested that epithelioid lesions tend to behave more often in a malignant fashion.[29,38,56] In the study of Newman *et al.* all malignant gastric tumours contained at least some foci composed of epithelioid cells, justifying their more cautious criteria for malignancy in epithelioid GISTs.[29]

Aside from oesophageal and colorectal tumours, which usually display fully developed features of smooth muscle differentiation (and therefore can be excluded, at least conceptually, from the GIST spectrum), significant differences in terms of outcome according to tumour site have only been observed in one study, in which the ten year survival reached 74% for gastric lesions, while it was only 17% for small bowel tumours.[32] In fact, during the last three years, a few studies have analysed prognostic factors in selected populations of tumours from specific sites, such as the duodenum but, again, have not been able provide definitive criteria for malignancy.[38,55,56]

Interestingly, some studies have shown a better prognosis in rare pediatric cases and in young adults, with long survival despite metastatic disease.[57] In fact, most of these patients appear to be affected by Carney's triad. In this syndrome, of which the genetic basis is still unclear, patients tend to develop gastric epithelioid 'leiomyosarcomas', functioning extraadrenal paragangliomas and pulmonary chondromatous hamartomas (which are sometimes clinically and radiologically misinterpreted as metastases from the gastrointestinal tumours). These tumours usually appear at a relatively young age and prolonged survival (more than 20 years) is commonly observed in the presence of metastases, even without surgical treatment.

### *Ploidy and proliferation markers*

Because of this imperfect separation between benign and malignant GISTs using conventional pathological criteria, as well as the subjectivity and/or interobserver variability in the evaluation of some of these parameters, ancillary techniques such as the evaluation of ploidy (by DNA flow cytometry or computerised image analysis) and proliferation markers were introduced with enthusiasm in the early 1990s. Most studies have suggested that DNA ploidy, determined by flow cytometry, was significantly correlated with histological grading and that aneuploidy was associated with decreased survival.[4,51,53,58] However, most of these authors compared tumour ploidy with malignancy defined either clinically but with a limited follow-up or defined only by histological criteria. A subsequent study, validated by 6 years median follow-up, demonstrated that ploidy lost its prognostic value in a multivariate model, which included mitotic count and the presence or absence of metastases at diagnosis.[52] Moreover, most of these studies included aneuploid cases that did not show clinical evidence of malignancy and, more importantly, a few patients with diploid tumours (even of small size), developed disseminated disease.[4,52] Because of this overlap, ploidy does not appear to be more discriminatory when applied in

**Table 1.** *Histological criteria for grading gastrointestinal stromal tumours*

| | | |
|---|---|---|
| Benign | 0–2 mitoses/30 HPF | spindle cell lesion, no atypia |
| | or | |
| | 0 mitoses/30 HPF | epithelioid lesion |
| Borderline | 2–3 mitoses/30 HPF | spindle cell lesion, mild plomorphism/hyperchromasia |
| | or | |
| | 3–4 mitoses/30 HPF | spindle cell lesion, no atypia |
| | or | |
| | 1 mitosis/30 HPF | epithelioid lesion |
| Malignant | $\geq$ 5 mitoses/30 HPF | spindle cell lesion, no atypia |
| | or | |
| | $\geq$ 3 mitoses/30 HPF | spindle cell lesion, frank pleomorphism/hyperchromasia |
| | or | |
| | $\geq$ 2 mitosis/30 HPF | epithelioid lesion |

individual cases, even if some correlation with prognosis can be shown at the statistical level.

Immunoreactivity for proliferating cell nuclear antigen (PCNA), and interphase nucleolar organiser regions (AgNOR) counts have also encountered variable success. While some authors failed to show any correlation between PCNA index and mitotic rate, tumour size or behaviour,[54] most other studies indicated a statistically significant relationship with survival in univariate analysis.[4,59–61] However, once again, the PCNA index, in multivariate analysis, did not appear to provide any improvement over the mitotic count in the prediction of metastatic spread[62] and it was shown (by Yu *et al.*) to be less reliable than histological grading using the scheme proposed in Table 1.[61]

Therefore, even if these 'modern methods' can be somehow correlated with survival or with the probability of developing metastases, from a statistical point of view, no significant advantage has been demonstrated over a careful mitotic count or histological grading. The clinical value of these techniques in individual uses is also limited, as is the case for traditional pathological criteria, by a degree of overlap between benign and malignant tumours. Although these proliferative indices may be helpful in some 'borderline' cases, and have been introduced in some classification systems,[60] their use is not warranted in the routine evaluation of GISTs at the present time.

*Practical recommendations*

It appears clear from the previous discussion that, at this point, published data available concerning phenotypic classification and prognosis are extremely controversial and somewhat confusing. Many reasons may have contributed to the heterogeneity of previous results. Most series are relatively small and, in statistical terms, do not include enough cases covering the different parameters assessed, as well as the different locations and immunophenotypes. Follow up is probably too short in the context of the biology of these neoplasms, given that late metastases, sometimes occurring after more than 20 or 30 years, are not uncommon. The definitions of malignancy, and in fact, the methodology have varied widely between studies. Most groups have stratified tumours according to histological criteria into categories (benign, uncertain potential, malignant) that they have then compared with clinical behaviour. In fact, a potentially more rational approach, consisting of classifying tumours according to their behaviour, after adequate follow-up, and then analysing their clinicopathological characteristics, has not been applied in any large series. Definitions of GIST have also varied widely between authors. Some authors have included typical cases of schwannomas, as well as conventional leiomyomas, thus introducing selection bias, while others have chosen to exclude all cases positive for muscle markers, or to exclude those which were negative for CD34.

Because of the unreliability of available criteria (when applied individually) in distinguishing tumours likely to behave in a benign or malignant fashion, multifactorial approaches have been attempted. A wide variety of prognostic schemes, including different parameters and varying according to tumour location, have been proposed. Actually, almost every single author has proposed his or her own classification scheme and, at the present time, none has proven superior to the others. For practical purposes, we personally use the criteria set out in Table 1. This table, established on the basis of personal experience and of relatively simple use, has proven useful in our daily practice and has appeared more reliable than any marker of proliferation.[61] However, other schemes, such as those presented by Suster in his review article,[63] have proven useful and, of course, they should also be considered.

It seems clear that larger series, with reproducible diagnostic and prognostic criteria, prolonged follow-up, including cases from various sites and covering a large number of parameters need to be collected. Until then, we believe that one should consistently use one scheme (such as that in Table 1), and that the inevitable uncertainty which presently exists concerning the behaviour of some GISTs, for example in the case of histologically benign but large tumours, should be expressed in the pathology report.

### Recent developments

*C-Kit* is a proto-oncogene encoding a transmembrane tyrosine-kinase receptor, KIT or CD117.[64] The interaction of this receptor with its ligand, the stem cell factor (SCF), has been shown to play an important role in the development of melanocytes, germ cells, mast cells and the interstitial cells of Cajal (ICCs).[65,66] The latter, which are located between the muscular layers of the gastric and intestinal wall in association with the myenteric plexus, are known to regulate the autonomous contraction of the gastrointestinal tract.[67,68] These cells are characterised immunohistochemically by dual immunopositivity for CD34 and CD117. Recently, the hypothesis that GISTs might differentiate towards an ICC phenotype, which had already been raised by Mikhael *et al.* on the basis of their immunopositivity for CD34,[36] has been supported by three immunohistochemical, ultrastructural and molecular studies.[9–11] These groups have demonstrated that immunoreactivity for CD117 was seen in 85–100% of GISTs while most other sarcomas or other neoplasms were negative. In addition, the sequencing of *c-Kit* complementary DNA revealed mutations in 5 of 6 cases from one of these series and our own personal experience with larger case numbers (Rubin *et al.*, unpublished data) is similar.

These studies included spindle cell and epithelioid neoplasms, confirming that they represent morphological variations of the same entity. CD117 positive tumours were also positive for CD34 in 72% of cases, while the (poorly specific) neural marker PGP9.5 was positive in about 70% of cases and smooth muscle actin was positive in 15–30% of cases.[10,31] Interestingly, all tumours showing immunohistochemical evidence of smooth muscle differentiation, i.e. desmin positivity and/or diffuse actin positivity, failed to stain for CD117, further justifying the separation of GISTs and conventional smooth muscle tumours in this anatomical location. These findings have led to a unifying concept, regarding GISTs as a morphologically (and immunophenotypically) heterogeneous group of tumours differentiating towards an ICC phenotype. The significance of CD34 negativity in a subset of these tumours, more often malignant in Sarlomo-Rikala's study,[11] is unclear, but a parallel has been made by Kindblom *et al.* with the variable immunophenotypes seen in the different subtypes of ICC.[10] GANTs, which, in our experience, are usually immunoreactive for CD117 but negative for CD34, could correspond to this subset of CD34 negative tumours and represent a distinctive group within the spectrum of GISTs.

As discussed above, the interpretation and comparison of most previous studies has been impaired by the lack of consistency in the definition of GISTs and therefore in the criteria for inclusion. The recognition of GISTs as a cohesive but phenotypically heterogeneous group of tumours, defined by the expression of *c-Kit*, and their more objective separation from other mesenchymal neoplasms (mostly true smooth muscle tumours) will certainly allow more reproducibility between studies and could help to refine our criteria for malignancy and/or prognostic factors. The expression of *c-Kit* also appears as a useful adjunct in the diagnosis of those GISTs arising in less usual sites, such as the mesentery, omentum or retroperitoneum, as well as in their separation from other intraabdominal neoplasms, such as desmoid fibromatosis for spindle cell lesions, and melanoma or metastatic carcinomas, which may be confused with epithelioid GISTs.

The similarity between the ultrastructural features of ICC and GIST, in Kindblom's study, not only provided further evidence for an ICC phenotype but also represents a rational explanation for the controversial results of early electron microscope studies. Ultrastructural characteristics shared by ICC and GISTs included incomplete features of myoid differentiation, such as networks of intermediate filaments, including occasional bundles of actin-type filaments or incomplete external lamina, as well as neurogenic features such as long interdigitating processes, occasional gap and desmosome-like junctions or synapse-like contacts.[10] This coexistence of myoid and neurogenic features probably explains the 'hesitations' between smooth-muscle and nerve sheath or neural differentiation in previous studies. Ultrastructural features of GANT (long cytoplasmic processes and synapse-like junctions with dense-core granules) were also part of this spectrum.[10,41]

*c-Kit* mutations had already been demonstrated in human malignant mastocytosis[69,70] and transfection of the mutant *c-Kit* complementary DNA into murine lymphoid cells has induced their autonomous growth.[9,71] The possibility that *c-Kit* might play some central role in tumour development or progression has recently received additional support, with the identification of a germline mutation in a family with multiple GISTs, in the same domain where mutations had been found in sporadic cases. Future investigations in such patients might therefore provide valuable information concerning the biology of GISTs. Losses in the long arm of chromosome 14, identified by one group using comparative genomic hybridization,[72] as well as the few reported karyotypic changes, including structural monosomies of chromosomes 14 and 22,[73,74] might also serve as the basis for further investigations. Study of cases arising in the setting of the Carney syndrome could also possibly generate genetic information that might help in better understanding sporadic cases.

### Conclusions

The two fundamental issues concerning GISTs, i.e. their phenotype and prediction of behaviour, had already been stressed by Stout and collaborators in their early description of stromal neoplasms of the gut in 1941. More than fifty years later, the probable differentiation of GISTs towards an interstitial cell of Cajal phenotype could well represent, at last, an elegant answer to the first question. Although the implications of *c-Kit* mutations in the pathogenesis of GISTs need to be further investigated, these molecular findings possibly represent the first step towards understanding the biology of these enigmatic tumours. Furthermore, the availability of reliable molecular and immunohistochemical signatures of GIST has already helped to clarify the extent of this spectrum, allowing more accurate distinction of GIST from other mesenchymal neoplasms of the gut, such as 'true' leiomyomas, leiomyosarcomas or nerve sheath tumours.

However, at the present time, the reliable distinction of benign from malignant GISTs remains a challenge. None of the multiple prognostic factors identified had proved to be reliable in the evaluation of individual cases and multifactorial approaches have thus far failed to improve significantly upon our morphological classification of GISTs. Hopefully, the recent findings relating to *c-Kit* mutations might improve our understanding of the enigmatic biology of GISTs in the near future and should improve the coherence of future clinical and pathological studies aiming at refining our prognostic criteria and classification schemes.

### References

1 Akwari OE, Dozois RR, Weiland LH, Beahrs OH. Leiomyosarcoma of the small and large bowel. *Cancer* 1978; 42:1375–84.

2 Van Steenbergen W, Kojima T, Geboes K, *et al.* Gastric leiomyoblastoma with metastasis to the liver. A 36 years follow-up study. *Gastroenterology* 1985; 89:875–81.

3 Ng EH, Pollock RE, Munsell MF, Atkinson EN, Romsdahl MM. Prognostic factors influencing survival in gastrointestinal leiomyosarcomas. *Ann Surg* 1992; 215:68–77.

4 Rudolph P, Gloeckner K, Parwaresh R, Harms D, Schmidt D. Immunophenotype, proliferation, DNA ploidy, and biological behavior of gastrointestinal stromal tumors: a multivariate clinicopathologic study. *Hum Pathol* 1998; 29:791–800.

5 Evans HL. Smooth muscle tumors of the gastrointestinal tract: a study of 56 cases followed for a minimum of 10 years. *Cancer* 1985; 56:2242–50.

6 Ng EH, Pollock RE, Romsdahl MM. Prognostic implications of patterns of failure for gastrointestinal leiomyosarcomas. *Cancer* 1992; 69:1334–41.

7 Bedikian AY, Valdivieso M, Khankhanian N, Benjamin RS, Bodey GP. Chemotherapy for sarcoma of the stomach. *Cancer Treat Rep* 1979; 63:411–4.

8 Plager C, Papadopoulos NE, Salem P, Benjamin RS. Adriamycin-based chemotherapy for leiomyosarcoma of the stomach and small bowel (Abstract). *Proc Annu Meet Am Soc Clin Oncol* 1991; 10:A1251.

9 Hirota S, Isozaki K, Moriyama Y, *et al.* Gain-of-function mutations of *c-Kit* in human gastrointestinal stromal tumors. *Science* 1998; 279:577–80.

10 Kindblom LG, Remotti HE, Aldenborg F, Meis-Kindblom JM. Gastrointestinal pacemaker cell tumor (GIPACT): gastrointestinal Stromal tumors show phenotypic characteristics of the interstitial cells of Cajal. *Am J Pathol* 1998; 152:1259–69.

11 Sarlomo-Rikala M, Kovatich AJ, Barusevicius A, Miettinen M. CD117: a sensitive marker for gastrointestinal stromal tumors that is more specific than CD34. *Mod Pathol* 1998; 11:728–34.

12 Golden T, Stout AP. Smooth muscle tumors of the gastrointestinal tract and retroperitoneal tissues. *Surg Gynecol Obstet* 1941; 73:784–810.

13 Appelman HD, Helwig EB. Gastric epithelioid leiomyoma and leiomyosarcoma (leiomyoblastoma). *Cancer* 1976; 38:708–26.

14 Ranchod M, Kempson RL. Smooth muscle tumors of the gastrointestinal tract and retroperitoneum. *Cancer* 1977; 39:255–62.

15 Martin JF, Bazin P, Feroldi J, Cabanne F. Tumeurs myoides intra-murales de l'estomac—considerations microscopiques a propos de 6 cas. *Ann Anat Pathol* 1960; 5:484–97.

16 Stout AP. Bizarre smooth muscle tumors of the stomach. *Cancer* 1962; 15:400–9.

17 Appelman HD, Helwig EB. Cellular leiomyomas of the stomach in 49 patients. *Arch Pathol Lab Med* 1977; 101:373–7.

18 Welsh RA, Meyer T. Ultrastructure of gastric leiomyomas. *Arch Pathol* 1969; 87:71–81.

19 Kay S, Still WJS. A comparative electron microscopic study of a leiomyosarcoma and bizarre leiomyoma (leiomyoblastoma) of the stomach. *Am J Clin Pathol* 1969; 52:403–13.

20 Knapp RH, Wick MR, Goellner JR. Leiomyoblastomas and their relationship to other smooth-muscle tumors of the gastrointestinal tract: an electron microscopy study. *Am J Surg Pathol* 1984; 8:449–61.

21 Mackay B, Ro J, Floyd C, Ordonez NG. Ultrastructural observations on smooth muscle tumors. *Ultrastruct Pathol* 1987; 11:593–607.

22 Mazur MT, Clark HB. Gastric stromal tumors: reappraisal of histogenesis. *Am J Surg Pathol* 1983; 7:507–19.

23 Weiss RA, Mackay B. Malignant smooth muscle tumors of the gastrointestinal tract: an ultrastructural study of 20 cases. *Ultrastruct Pathol* 1981; 2:231–40.

24 Yahigashi S, Kimura M, Kurotaki H, *et al.* Gastric submucosal tumours of neurogenic origin with neuroaxonal and Schwann cells elements. *J Pathol* 1987; 152:41–50.

25 Herrera GA, Cerezo L, Jones JE, *et al.* Malignant small bowel neoplasm of enteric plexus derivation (plexosarcoma). Light and electron microscopic study confirming the origin of the neoplasm. *Dig Dis Sci* 1984; 29:275–84.

26 Hjermstad BM, Sobin LH, Helwig EB. Stromal tumors of the gastrointestinal tract: myogenic or neurogenic? *Am J Surg Pathol* 1987; 11:383–6.

27 Hurlimann J, Gardiol D. Gastrointestinal stromal tumours: an immunohistochemical study of 165 cases. *Histopathology* 1991; 19:311–20.

28 Miettinen M. Gastrointestinal stromal tumors: an immunohistochemical study of cellular differentiation. *Am J Clin Pathol* 1988; 89:601–10.
29 Newman PL, Wadden C, Fletcher CDM. Gastrointestinal stromal tumours: correlation of immunophenotype with clinicopathologic features. *J Pathol* 1991; 164:107–17.
30 Ricci A, Ciccarelli O, Cartun RW, Newcombe P. A clinicopathologic and immunohistochemical study of 16 patients with small intestinal leiomyosarcoma. Limited utility of phenotyping. *Cancer* 1987; 60:1790–9.
31 Saul SH, Rast ML, Brooks JJ. The immunohistochemistry of gastrointestinal stromal tumors: evidence supporting an origin from smooth muscle. *Am J Surg Pathol* 1987; 11:464–73.
32 Ueyama T, Guo KJ, Hashimoto H, Daimaru Y, Enjoji M. A clinicopathologic and immunohistochemical study of gastrointestinal tumors. *Cancer* 1992; 69:947–55.
33 Pike AM, Lloyd RV, Appelman HD. Cell markers in gastrointestinal stromal tumors. *Hum Pathol* 1988; 19:830–4.
34 Franquemont DW, Frierson HF. Muscle differentiation and clinicopathologic features of gastrointestinal stromal tumors. *Am J Surg Pathol* 1992; 16:947–54.
35 Miettinen M, Virolainen M, Saarlomo-Rikala M. Gastrointestinal stromal tumors: value of CD34 antigen in their identification and separation from true leiomyomas and schwannomas. *Am J Surg Pathol* 1995; 19:207–16.
36 Mikhael AI, Bacchi CE, Zarbo RJ, Ma CK, Gown AM. CD34 expression in stromal tumors of the gastrointestinal tract. *Appl Immunohistochem* 1994; 2:89–93.
37 Monihan JM, Carr NJ, Sobin LH. CD34 immunoexpression in stromal tumours of the gastrointestinal tract and in mesenteric fibromatoses. *Histopathology* 1994; 25:469–73.
38 Tworek JA, Appelman HD, Singleton HP, Greenson JK. Stromal tumors of the jejunum and ileum. *Mod Pathol* 1997; 10:200–9.
39 Walker P, Dvorak AM. Gastrointestinal autonomic nerve (GAN) tumor: ultrastructural evidence for a newly recognized entity. *Arch Pathol Lab Med* 1986; 110:309–16.
40 Min KW. Small intestinal stromal tumors with skeinoid fibers: clinicopathologic, immunohistochemical and ultrastructural investigations. *Am J Surg Pathol* 1992; 16:145–55.
41 Lauwers GY, Erlandson RA, Casper ES, Brennan MF, Woodruff JM. Gastrointestinal autonomic nerve tumors: a clinicopathologic, immunohistochemical, and ultrastructural study of 12 cases. *Am J Surg Pathol* 1993; 17:887–97.
42 Shek TW, Luk ISC, Loong F, Ip P, Ma L. Inflammatory cell-rich gastrointestinal autonomic nerve tumor: expansion of its histologic spectrum. *Am J Surg Pathol* 1996; 20:325–31.
43 Antonioli DA. Gastrointestinal autonomic nerve tumors. Expanding the spectrum of gastrointestinal stromal tumors. *Arch Pathol Lab Med* 1989; 113:831–3.
44 Donner LR. Gastrointestinal autonomic nerve tumor: a common type of gastrointestinal stromal neoplasms. *Ultratstruct Pathol* 1997; 21:419–24.
45 Shanks JH, Harris N, Banerjee SS, Eyden BP. Gastrointestinal autonomic nerve tumours: report of nine cases. *Histopathology* 1996; 29:111–21.
46 Ojanguren I, Ariza A, Navas-Palacios JJ. Gastrointestinal autonomic nerve tumor: further observations regarding an ultrastructural and immunohistochemical analysis of six cases. *Hum Pathol* 1996; 27:1311–8.
47 Segal A, Carello S, Caterina P, Papadimitriou JM, Spagnolo DV. Gastrointestinal autonomic nerve tumors: a clinicopathological, immunohistochemical and ultrastructural study of 10 cases. *Pathology* 1994; 26:439–47.
48 Tsang WYW. Gastrointestinal autonomic nerve (GAN) tumors: an underrecognized group of gastrointestinal stromal neoplasms. *Adv Anat Pathol* 1994; 1:21–8.
49 Franquemont DW. Differentiation and risk assessment of gastrointestinal stromal tumors. *Am J Clin Pathol* 1995; 103:41–7.
50 Shiu MH, Farr GH, Papachristou DN, Hajdu SI. Myosarcomas of the stomach: natural history, prognostic factors and management. *Cancer* 1982; 49:177–87.
51 Cooper PN, Quirke P, Hardy GJ, Dixon MF. A flow cytometric, clinical, and histological study of stromal neoplasms of the gastrointestinal tract. *Am J Surg Pathol* 1992; 16:163–70.
52 Cunningham RE, Federspiel BH, McCarthy WF, Sobin LH, O'Leary TJ. Predicting prognosis of gastrointestinal smooth muscle tumors: role of clinical and histologic evaluation, flow cytometry, and image cytometry. *Am J Surg Pathol* 1993; 17:588–94.
53 El-Naggar A, Ro J, McLemore D, *et al*. Gastrointestinal stromal tumors: DNA flow-cytometric study of 58 patients with at least 5 years of follow-up. *Mod Pathol* 1989; 2:511–5.
54 Ma CK, De Peralta MN, Amin MB, *et al*. Small intestinal stromal tumors: a clinicopathologic study of 20 cases with immunohistochemical assessment of cell differentiation and the prognostic role of proliferation antigens. *Am J Clin Pathol* 1997; 108:641–51.
55 Brainard JA, Goldblum JR. Stromal tumors of the jejunum and ileum: a clinicopathologic study of 39 cases. *Am J Surg Pathol* 1997; 21:407–16.
56 Goldblum JR, Appelman HD. Stromal tumors of the duodenum. A histologic and immunohistochemical study of 20 cases. *Am J Surg Pathol* 1995; 19:71–80.
57 Persson S, Kindblom LG, Angervall L, Tisell LE. Metastasizing gastric epithelioid leiomyosarcomas (leiomyoblastomas) in young individuals with long-term survival. *Cancer* 1992; 70:721–32.
58 Kiyabu MT, Bishop PC, Parker JW, *et al*. Smooth muscle tumors of the gastrointestinal tract: flow cytometric quantitation of DNA content and correlation with histologic grade. *Am J Surg Pathol* 1988; 12:954–60.
59 Amin MB, Ma CK, Linden MD, Kubus JJ, Zarbo RJ. Prognostic value of proliferating cell nuclear antigen index in gastric stromal tumors. *Am J Clin Pathol* 1993; 100:428–32.
60 Franquemont DW, Frierson HF. Proliferating cell nuclear antigen immunoreactivity and prognosis of gastrointestinal stromal tumors. *Mod Pathol* 1995; 8:473–7.
61 Yu CCW, Fletcher CDM, Newman PL, Goodlad JR, Burton JC, Levison DA. A comparison of proliferating cell nuclear antigen (PCNA) immunostaining, nucleolar organizer region (AgNOR) staining, and histological grading in gastrointestinal stromal tumours. *J Pathol* 1992; 166:147–52.
62 Sbaschnig RJ, Cunningham RE, Sobin LH, O'Leary TJ. Proliferating-cell nuclear antigen immunohistochemistry in the evaluation of gastrointestinal smooth–muscle tumors. *Mod Pathol* 1994; 7:780–3.
63 Suster S. Gastrointestinal stromal tumours. *Semin Diagn Pathol* 1996; 13:297–313.
64 Chabot B, Stephenson DA, Chapman VM, Besmer P,

Bernstein A. The proto-oncogene c-kit encoding a transmembrane tyrosine kinase receptor maps to the mouse W locus. *Nature* 1988; 335:88–9.

65 Huizinga JD, Thuneberg L, Kluppel, *et al*. W/kit gene required for interstitial cells of Cajal, and for intestinal pacemaker activity. *Nature* 1995; 373:347–9.

66 Maeda H, Yamagata A, Nishikawa S, *et al*. Requirement of the c-kit for development of intestinal pacemaker system. *Development* 1992; 116:369–75.

67 Barajas-Lopez C, Berezin I, Daniel EE, Huizinga JD. Pacemaker activity recorded in interstitial cells of Cajal of the gastrointestinal tract. *Am J Physiol* 1989; 257:C830–5.

68 Langton P, Ward SM, Carl A, Norell MA, Sanders KM. Spontaneous electrical activity of interstitial cells of Cajal isolated from canine proximal colon. *Proc Natl Acad Sci USA* 1989; 86:7280–4.

69 Furisu T, Fujimura T, Tono T. Identification of mutations in the coding sequence of the proto-oncogene *c-Kit* in a human mast cell leukemia line causing ligand independant activation of *c-Kit* product. *J Clin Invest* 1993; 92:1736–44.

70 Longley BJ, Tyrrell L, Lu SZ, *et al*. Somatic *c-Kit* activating mutation in urticaria pigmentosa and aggressive mastocytosis: establishment of clonality in human mast cell neoplasms. *Nat Genet* 1996; 12:312–4.

71 Nishida T, Hirota S, Tanigushi M. Familial gastrointestinal stromal tumours with germline mutation of the *KIT* gene. *Nat Genet* 1998; 19:323–4.

72 Sarlomo-Rikala M, El Rifai W, Andersson L, Miettinen M, Knuutila S. Different pattern of DNA copy number changes in gastrointestinal stromal tumors, leiomyomas, and schwannomas. *Hum Pathol* 1998; 29:476–81.

73 Marci V, Casorzo L, Sarotto I, *et al.* Gastrointestinal stromal tumor, uncommited type, with monosomies 14 and 22 as the only chromosomal abnormalities. *Cancer Genet Cytogenet* 1998; 102:135–8.

74 Saunders LA, Meloni AM, Chen Z, Sandberg AA, Lauwers GY. Two cases of low-grade gastric leiomyosarcoma with monosomy 14 as the only change. *Cancer Genet Cytogenet* 1996; 90:184–5.
